# Traité complet de l'Hypochondrie

**Published:** 1844-10-01

**Authors:** 


I. TRAITÉ COMPLET DE L'HYPOCHONDRIE. Par *J. L. Brachet.* (Ouvrage Couronné par l'Academie Royale de Medecine. Octavo, pp. 736. Paris and Lyon, 1844. Baillière.

II. TRAITÉ PHILOSOPHIQUE DE MEDECINE PRATIQUE. Par *A. N. Gendrin,* D.M. Tome III. Octavo, pp. 675. Paris, 1843. Baillière.

THE name of *Brachet* deservedly stands very high in the present medical literature of France. It is now upwards of twenty years since he published an Essay on Hydrocephalitis; and, since that time, he has produced several esteemed works on various professional subjects—Memoirs on the Use of Opium in certain Inflammatory Diseases, and on Asthenia; Statistical Researches on the relative Numbers of Marriages and Deaths; an Elaborate Enquiry into the Functions of the Ganglionic System (1837); and a Practical Treatise on the Convulsions of Infancy. We are not therefore surprized that he has been the successful competitor for the prize offered by the Royal Academy of Medicine of France for the best memoir on the subject of Hypochondriasis. It is indeed a most elaborate and able work, and cannot fail to be recognised as a standard Treatise on the disease which it undertakes to describe. Dr. *Brachet* is a man of a calm reflecting mind, free from prejudices, and not under the dominion of any favourite doctrine. He does not profess to suggest any original views, or to propound any new theory, on a question that has vexed the ingenuity of medical speculators in every age; but he has applied himself to a much more useful subject, that of discussing the theories of others, collating and comparing them, one with another, and separating the results of actual observation from the delusive dreams of the imagination. His remarks throughout bear the impress of one who has seen much and read not a little; who has studied Nature for himself, but who has not neglected to make himself acquainted at the same time with the opinions of others, and more especially of the leading physicians of former times. Independently therefore of its own intrinsic merits, this work of our author deserves particular notice on another account;—inasmuch as it (and, we may add, that of M. Gendrin also) emphatically indicates a change in the spirit of medical literature that has sprung up of recent years, not only in France but also in our own country. Every one must have observed that there is a gradually increasing attention paid to the consultation and study of the writings of the old physicians, and that medical men, now-a-days, are not exclusively and altogether occupied with the plausible, but often most erroneous, explanations which have been given of the origin and treatment of maladies, since the beginning of the present century.

Something more, it is now generally acknowledged, is required, for the skilful management of disease, than a mere notation of the symptoms during life, and a knowledge of the appearances discoverable on dissection. The practitioner must learn to take a broad and comprehensive view of the various agents and influences which may have led to the development of existing diseases, and seek to appreciate the multitudinous, and often secret, circumstances that very materially modify their type as well as their duration and event. Now, there is not a member of the whole nosological catalogue, to which this remark is more truly applicable than that of Hypochondriasis; and it has been from the neglect of the precept here suggested that so many and so conflicting opinions have been held at different times as to its nature and mode of treatment. Our predecessors acted more wisely in this respect than we of late years have done. How frequent are the allusions in their writings to the modifying and often perverting influences of climate, season, locality and so forth, on epidemic and other diseases! The whole book of Hippocrates *de aere, aquis et locis* is full of them; and we find Celsus expressly saying, *aliud opus esse Romæ, aliud in Egypto, aliud in Galliâ.* Baglivi knew well the truth and importance of this, when he penned the simple but emphatic words: *Scribo in aere Romano.*

It is far from being an unprofitable labour, to study the literary history of a disease, and to collate and compare the writings of various authors in different ages and climates upon it. For, however fanciful and erroneous their theories respecting its origin and essence may be, there is always some good to be obtained from knowing the sentiments of observing and experienced men on such professional topics as are of a practical nature. It is for this reason that we propose, in the present article, to confine ourselves chiefly to that section of M. *Brachet's* work which gives an historical sketch of Hypochondriasis, derived from the writings of the most celebrated physicians from the earliest times down to the present age. As a matter of course we begin with the Father of Medicine himself.

In his second book *De Morbis,* Hippocrates obviously describes, in the following terms, the most prominent symptoms of Hypochondriasis, under the names of *morbus dessicatorius,* and *morbus ructuosus.*

. . . . . . *Neque sine cibo esse, neque cibum acceptum tolerare potest. Verum ubi quidem sine cibo manet, viscera sugunt, os ventriculi dolet, et aliâs alia vomit, et bilem, et salivam et acria; et postquam vomuit aliquantisper,*

*melius habere se videtur. At, ubi cibum accepit, ructus adsunt, et cum rubore exardescit, et semper multum se cacaturum esse putat. Ubi vero ea gratia desederit, flatus prodit et dolor caput habet, totum corpus velut aciculis pungi videtur, alias alia parte, et crura gravia sunt ac debilia, et consumitur ac debilis fit. Huic medicamentum bibendum dato, primum deorsum purgans, posteà superne, et caput purgato, et a cibis abstineat dulcibus ac oleosis et pinguibus, et ab ebrietatibus. Vomat autem a succis et a cibis, et pro anni tempore lac aut serum assininum bibendum præbens ; pharmacum insuper bibendum dato ; utro tandem magis opus esse tibi visum fuerit. Æstate et vere frigida lavet. Autumno autem et hyeme unguento utatur, et deambulet, et modice se exerceat. Si vero debilior sit quam ut exerceri possit, iter faciat. Et cibis frigidis ac alvum solventibus utatur. Et, si si venter non egerat, molle infusum per clysterem injiceto. Morbus hic diuturnus est ; et consenescentes, si ita futurum est, relinquit. Siu minus, commoritur.* In a subsequent passage in the same book, we find an emphatic allusion to the despondency and mental disquietude which so generally attend gastric and intestinal derangements : . . . . *Anxietudo ipsum invadit, lucem et homines refugit, tenebras amat, metus corripit, septum transversum exteriore parte intumescit, ad contactum dolet, expavescit, terriculamenta et formidanda cernit.* From these extracts it is abundantly obvious how well acquainted the old Coan was with the disease itself, if not with the name which was afterwards (from a mistaken theory) bestowed upon it. It has been generally alleged that Hippocrates attributed the symptoms of Hypochondriasis to the presence of *atrabilis* in the system. This, however, seems to be a mistake ; for there is not even a hint as to the *cause* of the malady in the passages from which we have quoted ; and it is only in a subsequent part of his writings that he treats of Hepatitis and other diseases of the liver, that we find an allusion to this supposititious humor.

Before proceeding with our selection of extracts, it is right to mention that M. *Brachet* groups and classifies the various authors, whom he quotes, under four heads, according as they have placed the seat of Hypochondriasis in 1, the Humours of the Body ; 2, the Abdominal Viscera ; 3, the Organic Nervous System ; or 4, the Brain and its attributes. Although objections may be very reasonably taken to this quarternary division,—inasmuch as many of the authors quoted have not held the single and exclusive doctrine imputed to them—we shall adhere, for convenience sake, to it, with one or two exceptions.

Galen—who is generally regarded as the founder of Humorism, in consequence of the great extension which he gave to its principles—did not fail to apply this doctrine to the explanation of the phenomena of Hypochondriasis. He attributed the disease to the blood becoming thick and viscid, and to the existence of *atrabilis* in the system. At the same time, he seems to have placed the actual seat of the malady—at least, at its commencement—*in* and *around* the stomach :—*cum igitur*, says he, *circa ventriculum inceperint accidentia, hypochondriacum flatuosumque morbum nominabimus.* The description, which he gives of it, is both longer and more lucid than had been given before his time. The Galenic view of the disease continued, with occasional modifications, to be the predominant one in the schools of medicine up to the epoch of the revival of letters. During this long period, the greater number of medical writers regarded the Liver and Spleen as the *foci,* whence arose those pernicious vapours which were considered as the proximate cause of all the hypochondriac's troubles.

Ætius pushed the humoral doctrine of the disease as far as possible; nevertheless he did not fail to observe that the earliest symptoms of the morbid process had their origin in the Brain. He supposed that the entire mass of the blood remained in a healthy condition, save and except that portion which is present in the brain: *Alio tempore, sanguine in toto homine illæso permanente, is, qui juxta solùm cerebrum est, alteratus;* and that a humoral vapour arose from the stomach to the head, the communication between the two being maintained by means of the pneumogastric nerves.

According to Avicenna also, the morbific matter which produces the disease, proceeds from the stomach and liver; the deranged state of which organs was regarded by him to be the primary and essential cause of all its phenomena.

Sennertus held a somewhat similar opinion; but he carried his views farther; for he maintained that the blood became corrupted by the unwholesome chyle that was generated in the digestive viscera, in consequence of the disorder of their functions. His definition of the disease is as follows: *Colluvies vitiosorum humorum, imprimis melancholicorum, sæpe etiam pituitorum et biliosorum in venæ portæ et arteriæ cæliacæ ac mesentericæ ramis, in hypochondriis, præcipuè sinistro, inter ventriculum et lienem, maxime iis locis ubi plures majoresque venæ portæ et arteriæ huic conjunctæ sunt.* The humorism of Sennertus is altogether more physiologically reasoned and argued than that of his predecessors.

The opinion of Vieussens coincided very nearly with that of Sennertus. Sometimes he made the disease depend upon an impure state of the blood; at other times upon obstructions of the bowels by vitiated chyle. But it was always some disordered state of the chylopoietic viscera that was viewed by him as the primary cause, and that which induced the unhealthy and corrupted state of the humours of the body.

In 1670, a sharp discussion took place between Willis—who, we shall afterwards find, was a zealous advocate of the nervous theory of Hypochondriasis—and Highmore, who followed in the wake of those authors we have been mentioning. In the reply of the latter to the former, we read, among the causes of Hypochondriasis, of the fibres of the stomach being feeble and relaxed; the digestion difficult and imperfect; and the chyle being badly elaborated, and mixing too soon with the blood, which becomes in consequence acrid and hot. The residue of the digestion, he says, remains too long in the stomach, and there it degenerates into a viscid phlegm, which subsequently ferments. The blood, also, being too fluid and flatulent, has a tendency to accumulate in the lungs and heart, and in these organs experiences a sort of effervescence which impedes the free play of their functions, and the easy descent of the diaphragm.

Lange supposed that the *materies* of the disease was a ferment generated in the spleen, and giving out a melancholic vapour, which was conveyed by the nerves to the stomach and other viscera. Stahl, in spite of his vitalism, attached so important an office to the vena portæ and its contained blood, that he may be classed at present among the humorists, more especially among those who had themselves dwelt much in their ætiological reasonings on the portal circulation, and who had designated this vessel, in consequence of the ills of which they supposed it was the vehicle, *vena portarum, porta malorum.* According to him, hypochondriasis consists in the congestion and irregular circulation of an over-viscid blood in this vein, and in the efforts which the vessel makes to rid itself of its superabundant contents. The windy atony he supposed to be the accidental cause of the symptoms—which are in part also attributable to the distention, or, as it used to be called, the *infarctus* of the meseraic and splenic vessels by the stasis of the blood within their cavities, and to the efforts of Nature to relieve herself of this partial obstruction. The opinion of Stahl, somewhat modified, was adopted by a great many writers. Thus we find that Perry imagined that the chief cause of hypochondriacal ailments is a thick, viscid, and melancholic state of the blood, which flows too languidly in the minute capillaries of the abdominal viscera. In consequence of this sluggishness of the circulation, the course of the animal spirits—the medium by which the soul and body were supposed to act and re-act upon each other—is more or less completely intercepted in the hypochondria, &c.—the necessary consequence of which is, that the mental energies themselves are feebly and irregularly exercised.

Boerhaave, always influenced by his mechanical theories, introduced them largely into all his ætiological speculations respecting hypochondriacal ailments. After admitting that, in some persons, the nervous system is unusually mobile and irritable, and that the nervous fluids are very easily disturbed, he supposes that a *cacochymia* of the blood is produced by the separation of its most fluid parts, giving rise to what he with the old writers calls *atrabilis.*

This fluid collects and accumulates in the abdominal viscera, becoming there more and more viscid and thick: *magis densa, tenax, immobilis reddita; inde sensim hic hærebit, accumulabitur, stagnabit; lienem, ventriculum, pancreas, omentum, mesenterium obsidens.* This is the Hypochondriasis *cum materia:* it was said to be *sine materiâ,* when the symptoms depend altogether on the exalted mobility of the nervous system in the hypochondria, and when they leave behind them no trace of pathological alteration.

The therapeutic precept, with which Etmuller closes his remarks on this disease, briefly but expressively announces his ætiological opinions: *cura ventriculum, et curasti hypochondriacum malum.* Riviere, although he classed hypochondriasis among the diseases of the spleen, attaches great importance to the operation of vitiated humours in the system: *Licet omnes partes,* says he, *hypochondriam constituentes in hoc morbo afficiuntur, lien tamen pars affecta præcipue censetur, cùm sit humoris melancholici receptaculum, ut non ineptè inter morbos lienis collocari valeat.* * * * * *Fit igitur affectio a melancholiâ præternaturali, quæ atrabilis nominatur, et ex aliis humoribus exustis et torrefactis generatur, præcipue sanguine, aut bili, aut melancholiâ naturali.*

Purcell, Silvius, Pitcairn, Mandeville, &c.—all took nearly the same view of the question, attributing the disease to a certain vitiation of the blood induced by the presence of acid and other crudities, generated within the stomach, in consequence of an unhealthy and cacochymic digestion. The second of these writers very judiciously remarks: *non male conjici*

*posse videtur sedem focumque hujus mali esse, si non ipsum ventriculum, ventriculo saltem vicinas partes.*

Stoll was the first who thought that a gouty humour had much to do with its production. Ludwig, although he classes hypochondriasis among the diseases of the lymphatico-serous system, traces back its remote cause to visceral disorder: *prima itaque hujus morbi semina in visceribus abdominalibus latent, postquam nimirum viscidus, salsus et crudus victus, inordinatè sæpius sumptus, una viis primis satis elaboratus.* Soon the blood begins to circulate more sluggishly in the vena portæ: *et infarctus varii connexum systema nervorum, paris vagi et intercostalis, ad spasmos deducunt, qui in abdomine quidem potissimum, sed et per consensum in aliis corporis partibus, observantur.*

About the commencement of the 17th century, a very notable change began to be introduced by the chemical physicians into the humoral pathology of hypochondriasis, as well as of many other diseases. Instead of *atrabilis* and corrupted humours and ferments being present in the system, we now read of nothing but of salts of tartar, acids, and alkalis predominating in the fluids. These foreign materials, generated by some process of fermentation or otherwise, and combined in various proportions, were believed to be the true *materies morbi* on most occasions.

It would much exceed the limits of the present article, if we attempted to follow out this part of our subject; but we cannot dismiss the humoral doctrines, to which we have alluded, without remarking that, however fanciful and absurd many of their positions are, the wise physician will not reject them entirely and without examination, more especially as there is an obvious tendency in the present day to the adoption of a modified Humorism. "Yes, without doubt," says our enlightened author, "the humours take and must take an active part in the production of various diseases. That is a truth which we firmly maintain. The difficulty is to explain how and in what manner they operate. Certain it is that none of those humoral principles, about which so much has been said, are cognisable by our senses; and indeed it is this very absence of actual proof that has given rise to the innumerable shades and varieties of pure Galenism. The foundation of the theory may be true; but the superstructure has generally been most visionary and ideal. Hence the absurdities of ferments, acrimonies, putrid and alkaline emanations, *atrabilis*, and all that host of crude and undigested humours, which have been supposed to be generated within the system."

We proceed now to notice a few of the most prominent authorities in favour of that doctrine which places the seat of hypochondriasis in some of the abdominal viscera—exclusively and independently of any alteration in the humours of the body.

The first writer we name in this list is Diocles of Carystos, whose description of the disease has been preserved to us by Galen.

*Oritur alius à ventriculo morbus, qui ab aliis melancholicus, ab aliis flatulosus, nominatur, quem, sumpto cibo maximè coctu difficili et caustico, sputum humidum idemque multum comitatur; item ructus acidissimi, flatus, æstus in hypochondriis; fluctuatio non illico, sed cum retinuerint; interdum ventriculi quoque vehementes dolores, qui nonnullis ad dorsum usque procedunt; concoctis deinde cibis, quiescunt. Mox aliis cibis ingestis eadum revertuntar*

*accidentia, quæ interdum jejunos, interdum etiam à cœna, molestant; atque evomunt crudos cibos et phlegmata subamara et calida adèo ut dentes torpedine afficiantur; flatuosas vocant affectiones. Suspicandum est plus caloris quam decet habere in venis quæ alimentum à ventriculo excipiunt, sanguinemque in ipsis crassiorem factum. Quippe constat has venas obstructas esse, vel hoc argumento quod alimentum corpori non distribuitur; sed ventriculo manet incoctum, cum ipsum meatus priùs susciperent, magnamque ipsius partem in crassum ventrem excernant, tum quod postero die vomitu rejiciatur utpote non digestus per corpus cibus, et quod calor, id quod secundum naturam est, excedat facile intelliges, tum ab æstu qui ipsis accidit, tum ab his quæ ingeruntur. Si quidem juvari videntur à refrigirantibus cibis qui videlicet calorem refrigerare extingueréque solent.* He subsequently remarks: *aiunt nonnulli os ventriculi, quod intestinis continuatur, ex hujusmodi passionibus inflammari, ac, ob inflammationem ipsam, et obstrui et prohibere ne alimenta statuto tempore ad intestina descendant.*

We are somewhat puzzled to understand how our worthy author, Dr. *Brachet,* after quoting this extract, should follow it up by saying: " These two observers (Hippocrates and Diocles) have confined themselves to enumerate the phenomena of the disease, without proposing any interpretation. Thus their descriptions always remain faithful and true to Nature, because they are not vitiated by the exigencies of a false theory." How are we to reconcile this praise, in the case of Diocles, with the obstruction of the veins of the stomach and the thickened and heated state of the blood in them of which we read, and to which the disease is ascribed?

Paulus of Egina considered Hypochondriasis to be the result of a burning heat, (for the word Φλογωσις, which he uses, does not mean *inflammatio* in its modern medical sense,) in the præcordial region, giving rise to a vapour which rose up to the brain: *tertia melancholiæ species est quam flatuosam et hypochondriacam, id est, præcordialem nominant, ex inflammatione præcordiorum circa stomachum obortâ, quæ aliquando auram quamdam pravam, et ad cerebrum, transmittunt.*

Paul Zacchias has expressed the same opinion in these words:—*Affectio hypochondriaca, quantum mihi videtur, nil aliud est quam calor naturalis hypochondriorum, unius vel scilicet plurium in ipsis contentarum partium, in calorem non naturalem, extraneum, et insolitum conversus.**

Forestus speaks of a *contumax imbecillitas ventriculi* as the most frequent cause of hypochondrisis. Baillon seems to have been much puzzled what to make of the pathology of the disease, and is more candid in his confessions than most writers; for he frankly admits his inability to determine the truth:—*Multi melancholiæ,* says he, *hypochondriacæ meminerunt, et hujus morbi nomen crebro usurpatum est; sed quid significet, quis locus affectus, cur ita appellatur, cùm non sit necesse melancholicum humorem in vitio esse, an intemperies sola sit, an morbus in conformatione, nondum satis definitum est.* When such a practitioner as Baillon writes thus, it should be an admonition to others not to be so hasty in solving the disputed question.

---

* The Arabian physicians, almost all of whom placed the seat of Hypochondriasis in the abdominal viscera, gave the disease the name of *Morbus Mirichialis*—from *mirach,* which signifies abdomen, epiploon, or mesentery.

Mead regarded Hypochondriasis as a disorder not of any one part of the body, but of the whole system. He however referred its origin chiefly to some morbid state of the abdominal viscera;—either of the stomach and bowels, or of the liver, or of the spleen and pancreas, or of the mesentery.

Prost, in his remarkable work, entitled *La Medecine Eclairée l'Ouverture des Corps*, laid the foundation of the gastritic doctrine which Broussais afterwards reproduced with so much eclat. After discussing the general pathology of nervous diseases, he thus expresses himself on the subject of Hypochondriasis; "This disease results from a change in the susceptibility of the nerves of the intestines, produced and kept up by a certain development of the arterial system in their mucous membrane: this disposition is accompanied by a more or less exalted degree of vitality in all the viscera of the abdomen." He subsequently repeats his belief, on more than one occasion, that this malady is owing to an increased susceptibility of the intestinal nerves and to their consequent re-action upon the Brain. Let us now hear what Broussais himself says upon the subject. In his celebrated Examen we find the following passage: "*Veritable causes of hypochondriasis:* The irritation of the chylopoietic viscera, even when the primary cause of the disease has been entirely of a moral nature, is that which opens the scene. It is this morbid state which by its influence depraves the intellectual functions, and gives rise to the host of pains, convulsions, and secretional changes, that are always present in a greater or less degree, and which, if not counteracted by appropriate remedies, gradually bring on a disorganisation of the viscera in which it is fixed, or so exhausts the vital energies as to bring on a fatal termination. Let your attention therefore be riveted on the great phenomenon of irritation of the stomach; and since it is this morbid state which causes all the suffering, engorgement, disorganisation, and also the convulsions and delirium, learn to subordinate every thing else to it." In a former part of the same work, he pithily defines Hypochondriasis to be "the effect of a chronic gastro-enteritis, that acts with energy on a brain predisposed to irritation." This doctrine, which for a good many years held almost undisputed sway in the French schools, is now fast disappearing from medical literature. Dr. *Brachet* is too enlightened an observer to be misled by its apparent simplicity. He objects to it on the two-fold ground, 1, That the local affection of the stomach, although it may be the determining cause of the disease, does not constitute it; for it cannot by itself produce a very important part of the phenomena of the disease, viz: the disorder of the intellectual energies; and 2, That, even although a gastric lesion be present in all hypochondriacal patients, this lesion is not necessarily, nor even frequently, of a genuinely inflammatory nature. M. *Georget* has clearly established these two points beyond all reasonable doubt; and subsequently M. *Barras*, in his excellent *Traité sur les Gastralgies et les Enteralgies*, has struck the last blow at the already tottering fabric of *gastritism*. To do however mere justice to *Broussais*, we should remember, says our candid author, that, in spite of all his predilections for a particular doctrine, he did not fail to recognise the necessity of the participation of the brain and of an indispensable anterior predisposition, in the pathological history of hypochondriacal diseases.

Before quitting this section of our subject, we gladly avail ourselves of the present opportunity to introduce the following remarks from the pen of *Cabanis*, on the influence of the state of the abdominal viscera on the manifestation of the mental phenomena.

"It is notorious," says that acute writer, "that in certain conditions of the internal organs, especially of the viscera of the abdomen, our faculties of perception and thought are always more or less influenced. The diseases of these parts change, disturb, and sometimes even entirely subvert, the habitual order of the sentiments and ideas. Extraordinary and bizarre appetites are developed; strange and hitherto-unknown images present themselves to the imagination; new affections lay hold of the will; and (what is still more remarkable) the mind often acquires more energy and elevation, and the heart is influenced by better and more affectionate feelings than it was heretofore. It is apparent therefore that much depends on the condition of certain viscera of the abdomen and the manner in which impressions act upon them, whether our ideas be bright or gloomy, and our feelings be kindly or the reverse. * * * * * Since the state of the abdominal viscera may entirely invert the order of the sentiments and thoughts, we cannot be surprised that diseases of these organs may induce insanity, &c."

Remarks, full of truthfulness and force!

We come now to notice briefly those authors—at least some of the most celebrated—who have considered Hypochondriasis to be a disease, primarily and essentially, of the general or organic nervous system, and who have therefore classed it among the Neuroses.

Willis is the first who distinctly and categorically announced this opinion. The nature of his studies, and the important office which he ascribed to the nerves in the performance of the functions of the body, and in the production of diseases, naturally led him to adopt this view of the case. He however mixed up some of the humoral notions of the day with his own particular doctrine; for we find him maintaining that the "point de depart" of the malady was in the Spleen, and that it was from this viscus that there arose certain morbific vapours which served to excite the spasms and contractions of the affected nerves: *Omnino a liene produci, quare ut plurimum vaporibus ab hoc viscere elevatis, et huc illue varië discurrentibus, ut sint partium nervosarum spasmi et contractiones.* The vitiated qualities, which the blood was supposed to acquire in the spleen, were the alleged cause of the explosion of the spirits *in cerebrum, indeque in genus nervosum.*

Sydenham adopted, with certain modifications, the opinion of Willis. In his language, Hypochondriasis is an *ataxia* (disorder or irregularity) of the animal spirits, which he believed to circulate within the nerves. *Pendent ergo*, says he, *affectiones istæ, quas in fœminis Hystericas, in maribus Hypochondriacas insignire libet, (quantum ego judico) a spirituum animalium αταξια, unde facto impetu in hanc illamve partem plus quam pro rata densi nimiique feruntur, spasmos uti et dolorem excitantes ubi in partes sensu exquisito præditas irruunt, atque organorum, tum ejus in quod se ingerunt, tum istius a quo abscedunt, functiones pervententes; cum utrumque ab hac tam iniquá partitione, quæ Naturæ oeconomiæ penitus adversatur, haud parum detrimenti capiat.* He combats the opinion that the disease has any necessary connexion with an alteration of the fluids, or with a morbid condition of any of the abdominal viscera. So decided was his opinion that the malady was purely nervous, that he regarded it as only one of the many forms of hysteria, and he described both maladies under the common appellation of the *vapours*—" a circumstance," says Dr. Brachet, " that we could not well account for, if we did not know that Hypochondriasis is so common in England that it is almost always associated with the hysterical affections of women."

The authority of Sydenham was long so great among medical men of all countries that his opinion was adopted by very many subsequent writers, some of whom carried their fancies respecting the course of the animal spirits, their stagnation, accumulation, obstruction, and explosion to a still more extravagant length. Thus, for example, Gorter says: " the proximate cause of Hypochondriasis appears to be a crude and acrid matter, which is thrown upon the nervous parts. This foreign matter, we may presume, becomes arrested in the capsule of the nerves, especially of those of the mesentery, stomach, spleen, and other viscera of the abdomen, whence it rises to the lungs, throat, and head."

Few writers have so frequently mentioned the disease of Hypochondriasis in their writings as Hoffmann; and this is probably the reason why his statements in different passages are not always consistent with each other. In one part he uses these words: *Radix mali hypochondriaci in ventriculo male affecto fixa est, in quo spasmi incipiunt, postea per propagines nervorum successivè serpunt, et consueta illa symptomata ingenerant.* Subsequently he places the seat of the disease in the nervous system : *non ultimum inter morbos generis nervosi spasmodicos vindicat sibi locum passio sic dicta hypochondriaca;* and he defines it thus : *est illa, si realem spectemus ideam, affectus primarum viarum, nominatim ventriculi ac intestinorum, spasmodicus flatulentus, ab inverso ac perverso illorum motu peristaltico natales suos mutuans; per consensum vero totum nervosarum partium systema in irregulares motus conjiciens et totam oeconomiam functionum perturbans.* It seems therefore, that, according to Hoffmann, the primary seat of the malady was in the nervous tunic of the alimentary canal.

The celebrated Lorry entirely adopted the opinion of Sydenham, and even carried it somewhat further than his master did; for he maintained the identity not only of Hypochondriasis and Hysteria, but also of these diseases and Melancholy : *hysterica et hypochondriaca affectio non differt a merâ melancholiâ nerveâ; easdem adhibent divisiones, diagnosim atque prognosim easdem, nec ullatenus curatione à se invicem abludunt.*

Whytt, who is generally acknowledged to be one of the ablest writers on the diseases of the nervous system, was of opinion that an over-sensibility and excessive weakness of the general system are the predisposing causes of the disease, which then becomes developed if a morbific humour that is circulating in the blood, or any powerful physical or moral influence, comes to act upon the nerves. An excellent commentary on this view of the subject is contained in the work of Pressavin. " We see," says this writer, " that the disease (he describes it under the name of *vapours)* is the effect of a peculiar idiosyncrasy of the nervous system, which becomes so mobile and sensitive that a very trifling cause is capable of exciting in it the most violent and irregular movements. . . . . The extreme delicacy of the nervous fibre is the sole cause of its excessive mobility and sensibility."

Lieutand expresses himself on the subject in the following terms: "The denomination of this spasmodic flatulent disease is derived from the *hypochondria*, which are supposed to be the chief seat of it. Conjectures, that seem to be well founded, render it probable that the immediate seat of the morbific action is in the veins which go to the formation of the vena portæ. . . . . However this may be, it appears manifest that the disease is purely spasmodic; that the nerves, being highly susceptible of irritation, play an important part in its production; and that the mind is quite as much, and perhaps even more, affected than the body. Hence it has happened that the term *hypochondriacal* has become almost a word of offence which medical men seek to avoid, bestowing the vague name of the *vapours* upon this affection."

Barthez, one of the very ablest professors and writers of the Montpelier School of Medicine, referred most of the symptoms of this as well as of other nervous diseases to some lesion of the Vital Principle. "The entire system of the vital powers," he writes, "is enfeebled by an alteration which has been introduced into the sensorial energies and their influence over the motor powers. When this general change of the vital forces has made great progress, it constitutes the malady known as the *vapours*, or as it is sometimes called, Neuropathy. Although very varied in its phenomena, it should not be confounded either with the hysteric passion, or with hypochondriacal melancholy, or with the nervous state which is limited to the organs of digestion, although each of these different affections may certainly co-exist along with it. . . . . The two elements of nervous diseases are the alteration of the sensory powers—these being either in excess or in defect—and the morbific influence of these on the motor energies."

The most able advocate, among the writers of the present century, of the nervous theory of Hypochondriasis, is unquestionably M. Louyer-Villermay, the title of whose work—*Traité des vapeurs ou maladies nerveuses, et principalement de l'hysterie et de l'hypochondrie*—sufficiently announces the ætiological opinions which the author holds. "It is not," says this very intelligent writer, "in the alteration or morbid lesion of the nervous system itself that the immediate cause of this Neurosis resides, but in an affection of the vital properties of the nerves of Nutrition. Hence it is that we generally recognise, as the seat of Hypochondriasis, the abdominal viscera—the stomach more especially—affected in their nervous system or vital properties, and particularly in their organic sensibility. In truth, we may see the simultaneous and primordial disturbance of all the organs, which serve to constitute the apparatus of digestion. To this disturbance there is added, from sympathy, a consecutive disorder of almost every organ in the economy, and consequently an exaltation of the general sensibility in the first instance; and in the second, a symptomatic affection of the moral and intellectual faculties."

Boisseau, in his *Nosographie Organique*, although he classes Hypochondriasis among the Neuroses, seems to believe that there is very often, if not generally, a sub-inflammation of the stomach present, and also an actual irritation of the encephalon, spinal marrow, and ganglionic system of nerves. "Is the irritation of the stomach," he asks, "inflammatory from the beginning? and is it present in all cases? It is difficult to answer these questions in a general manner: each patient must be attentively examined to determine the point. In every case, however, the disease terminates by declaring itself an intense chronic gastritis." He goes on to observe: "There cannot be Hypochondriasis without an affection of the brain; but this is often only secondary; for it frequently exists in persons whose reason is deeply affected." M. Roche, in his classic *Elements of Medico-chirurgical Pathology*, takes nearly the same view of the disease in question. According to him, the gastric membrane is the "point de depart" of all the morbid phenomena. Although classifying it among the neuroses of the mucous system, he insists much on the frequency of its supervening upon chronic Gastritis, and on the difficulty often experienced in distinguishing the one malady from the other.

Dr. James Johnson is of opinion that a special morbid sensibility of the digestive organs, more especially of the stomach, is the primary and chief cause of the phenomena of Hypochondriasis. This neurosis of the stomach is regarded as the essential malady, and all the concomitant accidents are viewed only as nervous effects resulting from the sympathetic re-action of the stomach on all the parts of the organism, in virtue of that diffusive property with which this organ is endowed.

Joseph Frank, in reference to this subject, says:—*Ad nos quod spectat, hypochondriasim pro morbo universi systematis nervosi, sese precipuè in gangliis abdominalibus, plexibus cardiacis et cerebro, modo cultro anatomico haud demonstrando, exserente, habemus.*"

According to Hufeland, the proximate cause of the disease is a pathogenic augmentation and anomalous condition of the sensibility of the nervous system, more especially of that of the stomach and other digestive organs; subsequently a re-action, and various new and extraordinary sympathies are developed. Notwithstanding the opinion here expressed, he has rather inconsistently placed it among the Vesaniæ or mental diseases.

Dr. Barras, the author of an admirable work *sur les gastralgies et les enteralgies*, has given a most accurate description of Hypochondriasis. He regards it as a special neurosis, that is often associated with gastralgia —which it is apt to induce, and of which it is often the consequence.

The nervous affection of the stomach becomes reflected, so to speak, on the encephalon; and this, becoming secondarily affected, gives rise to all the bizarre modifications expressed by the imagination.

As allied in some degree with the Nervous theory of Hypochondriasis, we may here notice the fanciful hypothesis advanced by Revillon in 1786, that this disease is induced by a certain modification of the electric fluid that, according to him, is always present in the animal body. He had been led to adopt this notion from finding in his own person how much the state of his feelings was influenced by the state of the wind and weather. His observations of his own feelings were doubtless quite correct, but his inferences were altogether fallacious; he mistook the *exciting*, for what medical men call the *proximate*, cause of the malady. His description, however, of the influence of the weather on his health is so graphic as to deserve especial notice:

*Venti ab oriente aut sicci aut rigidi, sæpius capitis affectiones, nempe oculorum, temporum, verticis, aut gravem aut perstringentem sensum excitare*

*solent; ubi alii, a meridie humidi et calidi, plerumque sensus tardant, tristitiam movent, totum corpus efficiunt hebes, languidum; imo pectoris molestias, æstum faciei vaporosum inferebant. Subita et magna ventorum discrimina subitas et magnas in affectiones differentias vehunt. Discrimen temperiei in atmospherâ multo magis sensum et motum variat in corpore quorumdam, quam mutet altitudines mercurii in barometro et thermometro.*

The comments of Dr. Brachet, on the nervous theories which we have rapidly reviewed, are contained in the following passage:—

"The doctrine, that views Hypochondriasis as a purely nervous affection, certainly approaches the truth more nearly than the opinions which we have hitherto examined. In truth all, or nearly all, the phenomena observed in the thorax, heart, stomach and other viscera of the abdomen, during the course of this variable disease, are fairly attributable to some disturbance of the function of innervation. Most of the facts, which we have collected together, may be adduced in favour of this view of the question. As a matter of course, we make no account of the various speculations, held at different times, respecting the animal or vital spirits, the nervous fluid, etherial principles, and so forth; all such fancies may be entirely kept out of view in our present enquiry. * * *

"The main defect of the nervous doctrine as to the cause of Hypochondriasis is, that it omits to account for a very material part of the phenomena of the disease; those namely which relate to the imagination and, its organ, the encephalon. * * * * The facts and reasonings adduced by M. Villermay, to claim for the nervous system the sole and exclusive prerogative of being the seat of the disease, are certainly not satisfactory; and it only requires to analyse them with attention to find weapons against his favourite doctrine. Simultaneously with the physiological lesion of the (organic) nervous system, there is almost always a not less important and constant lesion of the imagination and its organ. In most of the instances quoted by this gentleman, the cause acted directly upon the mind; in other words, it was of a moral, rather than of a physical, nature; not that we are prepared to maintain with certain writers (of whom we shall speak presently) that the primary and immediate seat of Hypochondriasis is always in the cerebrum. To this opinion we cannot assent. Often, very often, we see a patient, who is oppressed with grief, have no concern or apprehension whatsoever about the state of his health; it is the very last of his thoughts; and death indeed would be a relief to him. * * * * * Although the primary cause may act on the encephalon and its attributes, it is not by any direct or immediate operation on this organ that the disease of Hypochondriasis is induced. It first re-acts on the stomach and general nervous system, the mobility of which is excited and disturbed. It is to this nervous disturbance that the real and direct cause of the malady is to be traced."

But we must not anticipate too much; and therefore, before we say more on this subject, it will be right to draw the attention of the reader to a few of the authorities on the cerebral or mental side of the question.

Hippocrates, as we have already seen, alluded very emphatically to the existence of certain mental phenomena as constituting an essential part of the symptoms of Hypochondriasis; but he wisely abstained from fixing the seat of the disease in the brain, or indeed in any other part. Carolus Piso, or, as he is sometimes called, C. Lepois, was the first who distinctly advocated the cerebral pathology of the disease. "We believe," says he, "that all the symptoms falsely attributed to the stomach, uterus, and other organs, proceed from the head. It is this part, affected not in a sympathetic but in an idiopathic manner, that produces all the morbid movements felt over the entire body." This author imagined the existence of a *colluvies serosa juxta nervorum origines congesta,* to which he attributed the chief phenomena, both of hypochondriacal and of hysterical affections. Sauvage placed Hypochondriasis in the class of the Vesaniæ, or mental disorders. " This hallucination," he remarks, " proceeds from the over great attention of the mind to the preservation of the body, from the excessive love which we have for ourselves, and the nervous susceptibility thereby induced." Klockoff attributes the disease to the *tenor infirmatus medullæ cerebri.* Cullen classes it among the *adynamiæ,* the second order of his Neuroses : he ascribes the languor and depression of the spirits to an atony of the *sensorium.* Linnæus has placed it among mental disorders ; and Pinel has done the same, denominating it a neurosis of the cerebral functions. He however qualifies this opinion by telling us afterwards that " anatomical dissections have shewn that this malady is sometimes fomented by certain lesions of the abdominal viscera. * * Often, too, the evil depends upon a lesion of the functions of the nervous system, of which however there may remain no appreciable trace on dissection." Within the last 15 years, the cerebral or mental doctrine of Hypochondriasis has been warmly espoused by some clever writers in France, more especially by MM. Georget, Falret, and Dubois. These gentlemen maintain that the 'premier role' always belongs to the brain; that the 'point de depart' is always to be found in this organ; and that all the other phenomena are nothing but the effects of its re-action on the various organs of the body and their functions. Hence it has been proposed by them to designate the disease by the name of *Cerebropathy,* or *Encephalopathy.* The main arguments, adduced in favour of this doctrine, are—1, that the inducing cause of the malady is very frequently of a moral or intellectual nature ; as, for example, profound grief, excessive study, and so-forth ; and, 2d, that the leading phenomena are purely nervous and obviously very much under the influence of the brain, the centre of this system.

Let us hear what M. Brachet says on this matter.

" From a careful examination of all the facts and reasonings adduced by these gentlemen, we have no hesitation in expressing our opinion that the cerebral or mental theory of Hypochondriasis is not tenable in all its positions. It is quite right, in so far as it ascribes no little influence to the brain and its functions in the production of the disease; for, in truth, this malady cannot exist, if the encephalon be quite exempt from suffering or disturbance. Still it is perfectly true that neither does the morbid state in all cases commence with the brain, nor that the cerebral affection, in itself and essentially, constitutes it. The cause of the error, into which MM. Georget and Falret have fallen, arises from the circumstance that these distinguished physicians have been specially occupied with the study of insanity, and with the observation of insane patients; perhaps also from the known fact that Constitutional Hypochondriasis, which is almost always incurable, and is very different from the simple and accidental form of the disease, often exhibits the features of genuine Mania. If they had studied the malady in ordinary private practice, and not in the wards of a lunatic hospital or asylum, they would assuredly have modified their opinions. It must be only from allowing his mind to be occupied with a single train of ideas, to the exclusion of every other, that so acute an observer as M. Dubois could have carried his opinion so far as to assert that 'in the commencement of Hypochondriasis there is nothing material or organic or real, and that the only lesion is that of the understanding.' "

We have thus taken a rapid review of the most remarkable opinions that have been held by medical men of different ages, climates, and countries, respecting the nature, seat, or proximate cause of hypochondriacal disease. The diversity and discrepancy of these opinions may be traced to the besetting sin of professional writers, that of confining their vision to one part only of a subject, to the exclusion of every other : hence it is that they magnify and exaggerate one fact or object, and neglect and disparage all the rest. Now what is the real and simple truth ? M. Brachet will tell us :—

" It is quite true," says he, " that the functions of the stomach, liver, spleen, heart, &c., are more or less disturbed ; that the digestion is usually much deranged ; that an unhealthy chyle may be formed and supplied to the circulating fluids ; that the nerves are deeply affected ; and, finally, that the brain and the imagination are truly and essentially implicated. All this exists in Hypochondriasis ; there cannot be a doubt about the matter. But not one of these things taken singly, and apart from the rest, constitutes the disease in question. Every author, we verily believe, has truly seen what he has described ; but his mind, biassed either by the direction of his studies, or by the tone of the ruling doctrines of his day, has adopted one train of thought to the more or less complete exclusion of every other. Thus the opinions as to the nature and seat of Hypochondriasis have been *humoral* during the long reign of Humorism ; *organic*, when Solidism was dominant ; *nervous*, when so much importance was attached to the physiology of the nerves ; and at length *cerebral*, in the eyes of those men whose special attention has been engaged more particularly in the study of mental disorders. Now each and all of these doctrines are true ; at least partially so. The error lies only in their generalisation, in the attempt to reduce to a narrow spot phenomena whose sphere of exhibition is a wide circle. Not one of the theories that has been broached, however absurd and ridiculous it may now seem, was entirely destitute of considerable probability ; and for this reason— because they were all based on a series of observed facts and phenomena. It was only the extravagant extension, which was sought to be given to it, that rendered the opinion faulty, by making it too exclusive. It is to the multiplicity of opinions deduced from similar facts that *Galen* alluded to, when he said that in our economy there is nothing absolute, rigorous, and necessary, and that the same cause may produce different effects on different individuals : *Nihil in corpore animato planè est.*"

Having now sufficiently considered the much-vexed question as to the cause of Hypochondriasis, we proceed to a more satisfactory and important topic, that of its treatment. Fortunately on this point there is far less discrepancy and dissension :

*Concidunt venti, fugiuntque nubes,*
*Et minax* . . . . . *ponto*
*Unda recumbit.*

Hippocrates has not said much respecting treatment. From various passages of his writings we are led to infer that he recommended an occasional emetic, the moderate use of purgatives, along with a temperate diet and regular exercise. Celsus advises residence in the country, travelling about, gymnastic games—especially playing at ball and fencing— reading aloud, bathing, and friction after it—temperance in, but not abstinence from, venereal pleasures : *concubitus vero neque nimis concupis-*

*cendus, neque nimis pertimescendus est: rarus excitat corpus, frequens vero solvit.* Cælius Aurelianus insists chiefly on the necessity of amusing the mind and distracting it from its gloomy apprehensions. He lays down very particular rules; and even descends to mention the trifling circumstance of whitening the patient's chamber, to excite more cheerful feelings. He reprobates the use of strong medicines: *Vitandam probamus frequentem et variam medicaminum potationem, quæ fella deducere promittuntur, sive ventris fluxus, sive urinalia. Etenim sitis extenditur, et solidioris cibi fastidium duplicatur, et corporis fortitudo minuitur; atque cibi accepti corrumpuntur, et omnis corporis materia adulterio medicaminum deterior fit, cùm habitudo omnis fuerit immutata, quod facere videmus eos qui sæpissime ac jugiter absynthiam, aloem, et colocynthidem dari probaverunt.*

No writer, whether of ancient or of modern times, has discoursed more sagely on the treatment of Hypochondriasis than Galen. Fortunately, his therapeutics are not built upon, or deduced from, his humoral opinions; he rises above the spirit of system, and is at once practical and philosophical. His principal remedies were frequent baths, a juicy and nutritious diet, and all sorts of active exercise, *quæ non modo totum corpus exercent, verum etiam animum oblectare possint.* It was only in obstinate cases that he had recourse to purgatives and other evacuants to discharge the *atrabilis,* which was supposed by him to play so important a part in the induction of the malady.

Passing over the practice of Aretæus, Paulus Ægineta, and Ætius, for they seem to have suggested nothing new, we come to the epoch of the Arabian physicians, who, besides being indefatigable commentators of the writings of Hippocrates and Galen, introduced that host of polypharmic formulæ, known under the names of *elixirs, electuaries, pills, powders, troches, loochs, hiera, &c.* Many of their antispasmodic and other remedies are, although simplified in their preparation, still retained in practice, in spite of the opposition of the unbelieving philosophism of the present day.

Of all the Arabian writers, none has been so celebrated as Avicenna, who was long the oracle of medicine in the schools, and shared the homage of its professors with the old man of Cos and the physician of Pergamus. The following remarks by our author, in reference to the neglect of pharmaceutical remedies by his countrymen of late years, are so entirely consonant with the opinions which we have always expressed in this Journal, that we have much pleasure in introducing them here to the notice of our readers. "I do not desire," says he, "to revive that heap of bizarre, and often very ridiculous, formulæ; but I cannot help lamenting the other extreme into which we have fallen in the present day. It has landed us in such a famine, so to speak, of therapeutic resources, that people begin to enquire, when they read the clinical reports of some of our most able physicians, if medicine is now what it was before the time of Hippocrates—*the contemplation of death, or the necrology of a cemetery.* No where do we see any real efforts made to triumph over disease. Every exertion is made to determine most exactly the seat, extent, and form of a morbid alteration; and when they have succeeded in this object, they seem to fancy that everything possible has been done. Such knowledge, we readily acknowledge, is very necessary; but it is assuredly not all that is required of a medical man. He must not stop short at the diagnosis of a disease; he has then to proceed to a more important part still, the treatment of it. The old physicians acted in the very opposite manner; they neglected too much the diagnosis, and were altogether taken up with considering how to cure the disease they had to deal with. Their Materia Medica was certainly encumbered with far too many formulæ; and yet, we must confess, they often succeeded in obtaining a cure that could never be achieved by the use of gummed water! *To cure:* there is the end, the only philanthropic, end of medicine; and he, who succeeds the best in this glorious endeavour, deserves most highly of humanity."

Although Montanus, on one occasion, gave to a hypochondriacal patient the advice—which, by the bye, has often been very erroneously perverted into a precept of general application—*fuge medicos et medicamina, et sanaberis,* he expressly recommends the use of warm aperients, as rhubarb and turpentine, of regular exercise, friction of the extremities, &c. Platerus ordered his patients to take before their meals a powder composed of coriander and aniseeds, cannella, fennel and balm. Rhodius, following the example of Rondelet, cured a patient by trepanning him! Another favourite remedy with this gentleman was the application of the cautery over the line of the coronal suture! Sanctorius insisted much on the use of a vegetable diet and of regular exercise. Baillou gives the sound advice that ' hypochondriacal persons should eat little, but often, because their humours require to be continually renewed, in order that they may get rid of all acrimonies, and not generate any new ones by the fluids remaining too long within their vessels.' Zacchias trusted a great deal to the use of chalybeates, in conjunction with bathing. Burnet, in his *Thesaurus Medicinæ,* has collected together all the remedies that had been recommended by preceding writers: it is a compendium alike of regimen and therapeutics. Vieussens is in every respect a representative of the old humoral school. As he saw nothing in the disease but an impure and thickened blood and concrete state of the lymph, with engorgement of the abdominal viscera, his chief remedies were various depuratives, evacuants, bleeding, and subsequently tonics. Sydenham, in our author's estimation, was certainly not so judicious a practitioner, at least in the treatment of Hypochondriasis, as his " reputation aussi colossale," might justly have led us to expect. According to the English Hippocrates, the main indication is to restore to a healthy condition the blood which supplies the animal spirits; and as the *ataxia* or irregularity of these was supposed to induce, in course of time, an alteration in the circulating fluid, he recommended that bleeding and purgatives should be used in the first instance to lessen its quantity, and then that steel should be given to render tone both to the blood and to the animal spirits. If the patient was very weak, he began the use of chalybeates at once; and, during their administration, he exhibited no purgative medicines, but only gently soothing anodynes. He advised the application to the abdomen of a plaster of galbanum and castor; and, in some cases, administered medicated wines of gentian, absynthium, cinchona, &c. Horseback exercise and travelling were also favourite remedies with him.

Stahl, who considered that a stagnant state of the circulation of the vena portæ had so much to do with the disease, insists strongly on the good effects to be obtained from promoting a hæmorrhoidal discharge by the application of leeches to the anus, and the use of aloetic medicines. Hoffman advises a similar treatment, more especially at the time of the spring and autumnal equinoxes. He lays down the following four therapeutic rules to guide the practitioner: 1. To evacuate the crude and windy matters; 2. To dissipate the stagnant humours; 3. To appease the spasms; and 4. To fortify the nervous system. When health is once restored, he tells us thus how to preserve it: *tunc enim optimum præsidium est nullo uti remedio, sed præcipuum sanationis punctum in mutatione ætatis, aeris, vitæ generis, victusque consistere fide experientiæ compertissimum est.*

Dumoulin is reported to have said on his death bed, "I leave behind me three great physicians, diet, exercise, and water." Sauvages has laid down many judicious instructions, censuring those practitioners who trust exclusively either to mental or to physical remedies: the use of both ought always to be conjoined.

"If," says our candid author, "he has unnecessarily multiplied the species of Hypochondriasis—bilious, sanguineous, melancholic, pituitous, hysterical, asthmatic, phthisical, calculous, and tympanitic—he has shewn himself in matters of practice to be superior to many moderns, who seem to forget that the cure of the sick is the end and object of our science."

Of Whytt we are told that, while he accorded a proper degree of confidence to medicinal means, he never neglected to combine with their use a purely moral treatment. It is almost unnecessary to pursue this chronological notice of the therapeutics of Hypochondriasis any further; as we find an ever-recurring repetition of nearly the same remedies, till we reach the period when *gastritism* was the dominant genius of the schools. Fortunately this delusion has now nearly passed away, and physicians are beginning to imitate the practice of the good olden times, prescribing more for symptoms, and less for conjectural causes. Not many have adopted the *Cerebro-pathic* doctrines of Georget and Falret, who attach an almost exclusive, and therefore an undue, importance to moral and intellectual treatment. Not that we undervalue this part of hygienic regimen: far from it. We quite agree with Fontanelle that "a physician has almost as much to do with the imagination of his patients as with their lungs or their livers; and he must well understand how to treat their imagination, which demands particular specifics. A mere anatomist may dispense with eloquence; but a physician can scarcely do so. The one has only facts to discover and present to the eye; but the other, ever obliged to form conjectures on subjects often doubtful, requires also to base them on grounds that are solid, or which at least comfort and give assurance to the alarmed fancy. He must sometimes speak almost without any other object but to speak; as he has often the misfortune to deal with men at those very times when they are most feeble and childish. This puerility prevails principally among the higher classes, and more especially in that section of them who often require rather to be amused than to be cured. In general, if he (the physician) has not the gift of ready speech, he will need almost that of miraculous healing to compensate for the want."

This is all very well; but we need not say that soft words will not cure many cases of Hypochondriasis. Much may be done by physical as well as by moral regimen and treatment. The intestinal and urinary secretions must be carefully and regularly attended to. If the patient be subject to hæmorrhoidal discharge, this should generally be promoted rather than checked: *hæmorrhoides,* says Hippocrates, *melancholiam et lienis morbos curant.* Temperance and regularity should be peremptorily enjoined; moderation in sexual pleasures also is very necessary: *quibus nervi dolent, Venus nocet.* One of the most useful of all remedies is manual employment and bodily exercise. M. Reveillé-Parise tells us of a patient who had been recommended to take a sea-voyage for the cure of his malady. A friend had very considerately recommended him to read the philosophic works of Seneca on board, with the view of distracting and comforting his mind: "mais quel pauvre medecin de l'ame et du corps, en comparaison du travail des pompes et de cabestan!" he himself frankly confessed to his physician on his return.

Travelling, and change of air and scene, have been lauded since the days of Hippocrates: *in morbis longis solum mutare debent,* says the old Sage; and Baglivi expresses the same thought in these words: *evenit morbos peregrinatione desinere, qui antea nulli medicamini cedebant.* This most accomplished physician—whose works we strongly recommend to the Sydenham Society to reprint—in another passage thus beautifully points out the soothing and sanative influences of a judicious hygienic regime on hypochondriacal patients: *Et licet talium hominum morbi primo aspectu perniciosi et incurabiles videntur, sanari tamen solent facile non quidem per nimiam remediorum copiam, sed aut per grata amicorum colloquia, aut per honesta ruris oblectamenta et equitationes frequenter, aut tandem per vivendi normam a sagaci medico institutam.*

In all cases, it is very necessary to attend to the state of the skin, to promote perspiration, and encourage an active circulation on the surface: hence the importance of shower or plunge bathing, and brisk friction with the horse-hair gloves, or a flesh-brush, &c. The intimate connection between the cutaneous and the digestive organs has long been understood; and their diseases are often observed to alternate with each other.

Hypochondriasis has been known to cease very quickly, or even suddenly, on the breaking out of a cutaneous eruption. Reil says: *nam subito exanthemata, ulcera &ca. oriuntur, et eodem momento morbus nervosus præsens cessat.* Lorry expresses the same thought: *sæpe scabies, impetigo, herpes erumpentes sanitatem retulere;* and Heine makes a similar remark in these words: *attulit sæpe curationem scabies fœda, aut varix numerosa ingens enata, valde tumentium fluxus hæmorhoidum, atrabilis per superiora et inferiora rejectio.* A sharp attack of the gout, as Stoll has remarked, has often been known to dissipate not only hypochondriasis, but even melancholy and mania: *subortâ arthritide sæpe curantur.*

It has been remarked that hypochondrical patients are more than usually exempt from contagious and epidemic disorders: *Hypochondriaci,* says one of the old writers, *morbis contagiosis et epidemicis rarius corripiuntur; nervi, ad spasmos efficiendos activi, sensu pro contagio carent; si quondam efficiuntur, hypochondriasis cessat.*

There are many other topics connected with the treatment of Hypochondriasis that we could wish to have alluded to, if our space had permitted. One word respecting the use of sedatives. Medical practitioners are often too timid in the administration of this class of medicines in many nervous diseases. If no sharp pain be present, and if the patient sleep moderately well, it is certainly better to abstain from using them; but let it be remembered that *nihil molestat sicat dolor*, and that a restless night will often suffice to throw a patient back farther than a week's suffering during the day-time. A mild soporific—which should generally be combined with a gentle aperient—at bed-time will often much promote the cure.

We conclude with the closing paragraph—to the sentiments of which we fully respond—of M. Brachet's excellent work.

"Let the physician never forget that he has to deal, at one and the same time, with a sick body and a diseased mind. Let him therefore not fail to make use of moral and physical treatment simultaneously, and not trust to either exclusively. Let the careful study of each patient direct the selection of the remedies to be employed in his case. Let him in general avoid the use of all very potent or drastic medicines and have recourse to them only in certain peculiar cases, and even then with the greatest reserve. Let him warn his patient against an avidity for a variety of remedies. The exaggerated advice of Montanus—*fuge medicos et medicamina, et sanaberis*—would be less hurtful than an indiscriminate poly-pharmacy. Rather than let him fall into the hands of charlatan medicasters, it would be much better to inculcate upon him the memorable couplet of Arnaud de Villeneuve,

*Si tibi deficiant medici, medici tibi fiant*
*Hæc tria: mens hilaris, requies moderata, diæta.*"

It only remains for us to say a few words about M. Gendrin's new volume. It gives a most elaborate, but often too a most wearisomely minute, history of Dyspeptic Complaints. If a separate volume is to be devoted to each disease or groupe of diseases, how many will be required to complete this Philosophical Treatise? We cannot say how philosophers may like this multiplication of books; as critics we must enter our protest against it, as most unnecessary and unprofitable.

Although we say this, we must acknowledge that there is a great deal of curious and valuable matter in the present volume.

